# Does Oxidative Stress Management Help Alleviation of COVID-19 Symptoms in Patients Experiencing Diabetes?

**DOI:** 10.3390/nu14020321

**Published:** 2022-01-13

**Authors:** Alok K. Paul, Md K. Hossain, Tooba Mahboob, Veeranoot Nissapatorn, Polrat Wilairatana, Rownak Jahan, Khoshnur Jannat, Tohmina A. Bondhon, Anamul Hasan, Maria de Lourdes Pereira, Mohammed Rahmatullah

**Affiliations:** 1 School of Pharmacy and Pharmacology, University of Tasmania, Private Bag 26, Hobart, TAS 7001, Australia; alok.paul@utas.edu.au; 2 Department of Biotechnology & Genetic Engineering, University of Development Alternative, Lalmatia, Dhaka 1207, Bangladesh; rownak86@hotmail.com (R.J.); jannat.koli.22@gmail.com (K.J.); afrozebondhon@gmail.com (T.A.B.); anamulhasanoris@gmail.com (A.H.); 3 Institute for Health and Sports, Victoria University, Melbourne, VIC 3011, Australia; md.hossain18@live.vu.edu.au; 4 World Union for Herbal Drug Discovery (WUHeDD) and Research Excellence Center for Innovation and Health Products (RECIHP), School of Allied Health Sciences, Walailak University, Nakhon Si Thammarat 80160, Thailand; tooba666@hotmail.com (T.M.); nissapat@gmail.com (V.N.); 5 Department of Clinical Tropical Medicine, Faculty of Tropical Medicine, Mahidol University, Bangkok 10400, Thailand; 6 CICECO-Aveiro Institute of Materials & Department of Medical Sciences, University of Aveiro, 3810-193 Aveiro, Portugal; mlourdespereira@ua.pt

**Keywords:** COVID-19, diabetes mellitus, oxidative stress, kidney damage, antioxidant

## Abstract

Severe acute respiratory syndrome (SARS)-CoV-2 virus causes novel coronavirus disease 2019 (COVID-19) with other comorbidities such as diabetes. Diabetes is the most common cause of diabetic nephropathy, which is attributed to hyperglycemia. COVID-19 produces severe complications in people with diabetes mellitus. This article explains how SARS-CoV-2 causes more significant kidney damage in diabetic patients. Importantly, COVID-19 and diabetes share inflammatory pathways of disease progression. SARS-CoV-2 binding with ACE-2 causes depletion of ACE-2 (angiotensin-converting enzyme 2) from blood vessels, and subsequently, angiotensin-II interacts with angiotensin receptor-1 from vascular membranes that produce NADPH (nicotinamide adenine dinucleotide hydrogen phosphate) oxidase, oxidative stress, and constriction of blood vessels. Since diabetes and COVID-19 can create oxidative stress, we hypothesize that COVID-19 with comorbidities such as diabetes can synergistically increase oxidative stress leading to end-stage renal failure and death. Antioxidants may therefore prevent renal damage-induced death by inhibiting oxidative damage and thus can help protect people from COVID-19 related comorbidities. A few clinical trials indicated how effective the antioxidant therapy is against improving COVID-19 symptoms, based on a limited number of patients who experienced COVID-19. In this review, we tried to understand how effective antioxidants (such as vitamin D and flavonoids) can act as food supplements or therapeutics against COVID-19 with diabetes as comorbidity based on recently available clinical, preclinical, or in silico studies.

## 1. Introduction

The novel coronavirus disease 2019 (COVID-19) is caused by severe acute respiratory syndrome CoV2 (SARS-CoV-2) virus and can be associated with infected patients with various comorbidities such as diabetes, hypertension, and cardiovascular disorders. Studies show that the viral infection triggers severe clinical symptoms and mortality with people experiencing comorbidities such as diabetes, cancer, and heart and lung disorders. Importantly among these people, diabetic patients experience the most severe clinical symptoms that cause the highest proportional death than non-diabetic patients after SARS-CoV-2 infection [1,2]. Along with diabetes, old age, congestive heart failure, smoking, β-blocker use, presence of bilateral lung infiltrates, elevated creatinine and severe vitamin D deficiency” are significant cause of mortality in COVID-19 patients [3]. In addition, high plasma lactate dehydrogenase level, a marker of oxidative stress, and advanced age 70 years or above) showed increased mortality, anxiety, and severity of COVID-19 symptoms in the clinic [4,5,6]. Several questions need to be answered to understand the pathophysiological connections between COVID-19 and diabetes mellitus, which leads to an increase in fatalities. Approximately four different pathogenesis are involved in SARS-CoV-2 infection, such as activation of the renin-angiotensin (RAS) pathway, oxidative stress, excess cytokines release, and dysfunction of endothelium. COVID-19 develops after SARS-CoV-2 entry in host’s cells and RAS activation with oxidative bursts [7,8]. In this article, we give some insights on common features between diabetes and COVID-19-induced kidney damage and discuss the implications of increased oxidative stress in the process, which may help improve patient prognosis.

## 2. Is Oxidative Stress a Major Cause of Diabetes-Induced Kidney Damage?

Diabetes is one of the most common metabolic disorders influenced by several factors such as age, sex, ethnicity, genetic factors, and pregnancy and appears as a comorbidity with obesity, cardiovascular diseases, atherosclerosis, renal failure, cancer, and many other chronic diseases [9]. People with diabetes show an impaired function of insulin (insulin resistance) and therefore need an increased amount of insulin than β cells (in the pancreas of a person) can produce. As a result, the presence of higher blood glucose in the bloodstream is observed. It has been postulated that diabetic nephropathy develops due to localized oxidative stress, where the key initiator may be increased mitochondrial production of reactive oxygen species (ROS) arising from hyperglycemia and leading to various renal disorders [10]. Diabetic nephropathy is present in almost one-third of Type 1 and Type 2 diabetic patients [11]. Diabetic neuropathy, nephropathy, and retinopathy can arise from oxidative stress-induced complications in diabetes mellitus along with a host of other disorders like coronary artery disease [12].

**Figure 1:**
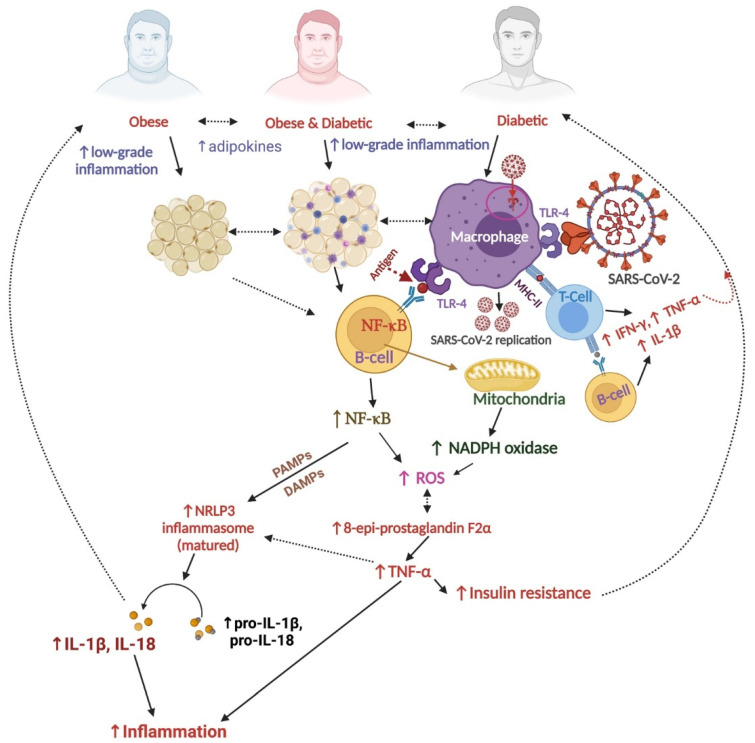
Correlation between SARS-CoV-2, oxidative stress, diabetes, and obesity. Abbreviations: ↑: increase; ROS, reactive oxygenated species; TNF-α, tumor necrosis factor α, TLR, Toll-like receptor; IL, interleukin; NADPH, nicotinamide adenine dinucleotide phosphate oxidase; IFNγ, gamma interferon; NF-κB, nuclear factor-kappa B; NLRP3, nucleotide-binding oligomerization domain-like receptor family pyrin domain containing 3 inflammasome; PAMPs, pathogen-associated molecular patterns; DAMPs, damage-associated molecular patterns; MHC-II, major histocompatibility complex class II. (The figure was made with www.biorender.com, accessed on 13 December 2021).

Diabetes is considered to be one of the major indicators for severe COVID-19 prognosis, as more diabetic patients (diabetes type-2 is mainly evident, with limited evidence from diabetes Type-1) showed severe COVID-19 symptoms and deaths after exposure to SARS-CoV-2 virus [1,13,14,15]. A meta-analysis concluded that the diabetic patients showed a 200% increased probability of death with severe COVID-19 symptoms than non-diabetic patients [16]. Importantly, Toll-like receptor 4 (TLR4) is responsible for initiating diabetes by expressing the transcriptional factor nuclear factor-kappaB (NF-κB) and the enzyme nicotinamide adenine dinucleotide phosphate (NADPH) oxidase to produce ROS, which also induce activation of endothelial nitric oxide synthase (eNOS) and xanthine oxidase enzymes [17]. Together these enzymes produce excess ROS and can be the causative agent(s) for diabetes-like diseases [18]. Another recent study reported that presence of diabetes mellitus type 1 results in increased morbidity and mortality rates during coronavirus (COVID-19) disease [13]. Diabetic patients displayed higher cell counts of leukocytes and neutrophils in their blood during admission with comparatively severe COVID-19 symptoms than non-diabetic patients. The diabetic patients also required more antibiotic therapy and artificial ventilation, but still resulted in more deaths during their stay in the healthcare facilities in China [1]. Oxidative stress also causes decreased use of glucose by muscles and adipose tissues. An increase of 8-epi-prostaglandin F2α, an oxidative stress indicator, is positively correlated with insulin resistance [19] (Figure 1). Insulin resistance is also thoroughly interrelated with inflammation as a preclinical study showed increased tumor necrosis factor α (TNF-α) from adipose tissues of obese and diabetic animals), a proinflammatory cytokine that can cause insulin resistance; suppression of TNF-α helps recovery of insulin resistance [20] (Figure 1). NLRP3 (nucleotide-binding oligomerization domain-like receptor family pyrin domain containing 3), a polyprotein complex inflammasome found in macrophages, is also responsible for causing diabetes and the release of inflammatory cytokines. NLRP3 is stimulated by the activation of NF-κB (nuclear factor-kappa B, which is triggered by TNF-α) and causes the secretion of proinflammatory cytokines pro-IL-1β and pro-IL-18 (Figure 1). NLRP3 matures by PAMPs (pathogen-associated molecular patterns) and DAMPs (damage-associated molecular patterns) or lipopolysaccharides. The maturation of NLRP3 causes the release of cytokines such as IL (interleukin)-1β and IL-18 and inflammation in the body [21]. Adipose tissues mainly produce inflammatory biomarkers such as TNF- α, and macrophages and other immune cells are partially responsible for insulin resistance. Type-2 diabetic patients show increased inflammatory cytokines and autoimmune responses in the pancreatic islet cells and can cause insulin resistance and decreased insulin secretion, although the whole mechanism is not yet clearly understood [22]. Oxidative stress, insulin resistance, inflammation, and kidney cell damage are interrelated and part of a chronic pathophysiological mechanism.

## 3. What Is the Clinical Evidence on the Relationships between COVID-19 and Diabetes?

Some studies suggest that the COVID-19 vaccination should be prioritized in diabetic patients (both type 1 and 2) as they have a poorer prognosis with COVID-19 compared to COVID-19 patients without diabetes [23,24]. A recent randomized clinical trial on the Scottish population (a population cohort study) in the first wave found increased severity in COVID-19 symptoms and admitted for fatal and critical care units for treatment with diabetes compared with those without diabetes [25]. The overall odds ratio for diabetes was 1·395, calculated against patients without diabetes, which means diabetes was strongly positively correlated with the severity of COVID-19 patients. Noticeably, the odds ratio for the severity of Type-1 diabetic patients were much higher than Type-2 diabetic patients [25]. Another RCT with children with Type-1 diabetes in the US suggested that preintervention and social support improved the children to manage COVID-19 pandemic-related stress and depressive symptoms for the children and their parents [26].

Another ongoing RCT with COVID-19 patients introduced “telemetric continuous glucose monitoring” for patients with positive diabetes suggested remote glucose monitoring may provide similar results to conventional finger-prick test (*n* = ~36 each group) but better outcomes as it needs less exposure of healthcare workers and fewer risk of cross-contaminations or reinfections [27].

A further RCT in Taiwan tried to educate and guide patients with diabetes Type-2 to maintain their health during the COVID-19 pandemic and found that the health-related coaching helped keep patient’s glycosylated hemoglobin (HbA1 c) levels under control; they maintained physical exercises, and reduced eating out [28].

A systematic review investigated the relationships among periodontal diseases, diabetes, and COVID-19 and indicated that hyperglycemia (e.g., diabetes) might increase the possibilities of periodontitis development and influence excessive expression of angiotensin-converting enzyme 2 (ACE-2) in periodontal tissue of diabetes Type-2 patients [29]. In addition, the excessive ACE-2 can favor the SARS-CoV-2 virus to develop COVID-19 [29]. Therefore, periodontal diseases or diabetes type 2 can potentially influence the development of COVID-19 symptoms and go for mild to severe form depending on the physiological and pathological conditions of the patients. However, no proper randomized clinical trials are evident to date proving this relationship.

## 4. How Can SARS-CoV-2 Damage the Kidneys?

SARS-CoV-2 enters the host body interacting with the angiotensin-converting en-zyme-2 (ACE-2), which is present in multiple organs, mainly kidneys, lungs, testis, breast, heart, and gastrointestinal systems [30]. SARS-CoV-2 interacts with angiotensin-converting enzyme 2 (ACE2) and causes an increase of angiotensin-2 in tissues that activates CD8+ and CD4+ T-lymphocytes macrophages and NK cells and releases pro-inflammatory and inflammatory cytokines such as IL-1β, IL-2, IL-4, IL-17, IL-21, and IFNγ (gamma interferon) [31]. SARS-CoV-2 interaction with TLR4 in macrophages can also activate major histocompatibility complex (MHC) class II molecules and thus in-crease T-cells- and B-cells-mediated secretions of proinflammatory cytokines (IL-1β, IFNγ, and TNF-α) (Figure 1). The released inflammatory cytokines from the lungs, kidneys, or elsewhere in the body because of SARS-CoV-2 infection, are transported through the bloodstream that causes quick acute inflammation in the capillaries of kidneys, lungs, heart, and all major organs.

ACE-2 receptor is expressed mainly in proximal tubular epithelial cells in both diabetic and healthy kidneys, but diabetic patients express higher ACE-2 receptors in their pancreatic islets than normoglycemic patients [32]. In COVID-19 patients, ACE-2 receptor containing proximal tubular epithelial cells has been detected in urine samples, suggesting a common infection pattern of SARS-CoV-2 in patients with diabetes [33]. Importantly, overexpression of ACE-2 receptors in the proximal tubular epithelium of diabetic patients may cause severe SARS-CoV-2 associated clinical symptoms and damage to kidneys as microscopic examination of COVID-19 infected kidneys showed proximal tubular injury and acute tubular necrosis [34]. Another study indicated that acute injury in the kidney is responsible for the increased morbidity and mortality of SARS-CoV-2 infected patients [35].

ACE-2 binding of SARS-CoV-2 causes depletion of ACE-2 receptors that may facilitate the binding of angiotensin-II with angiotensin receptor-1 from blood vascular membranes that produce NADPH (nicotinamide adenine dinucleotide hydrogen phosphate) oxidase, oxidative stress and cause constriction of blood vessels, platelet aggression, the release of proinflammatory cytokines (i.e., inflammation), and increase the severity of the infection [36,37]. SARS-CoV-2 induced severe infection also causes a high neutrophil/lymphocyte ratio that generates increased reactive oxygen species levels. The oxidative stress further induces platelet dysfunction and tissue damage in the lung, kidney, and other major organs [38].

In a cross-sectional study conducted with 50 COVID-19 patients in Nigeria, oxidative stress marker, 8-isoprostaglandin F2α, was found to be significantly higher (*p* = 0.049); on the other hand, malondialdehyde (MDA) was lower (*p* < 0.001) in COVID-19 patients than controls. The authors further concluded that COVID-19 infections and other comorbidities such as diabetes, malaria, and hypertension increased the risks of developing oxidative stress [39]. Furthermore, increased oxidative stress could be responsible for “amplifying and perpetuating the cytokine storm, coagulopathy, and cell hypoxia” in COVID-19 patients [40]. Oxidative stress has also been described as a ‘key player’ in the pathogenesis, severity, and mortality risk in SARS-CoV-2 infections [41]. A systematic review and me-ta-analysis showed that acute respiratory distress syndrome development in COVID-19 patients accelerated the development of acute kidney injury (AKI) and higher mortality rate [42].

## 5. Synergistic Kidney Damage and Morbidity Due to COVID-19 and Diabetes

**Figure 2:**
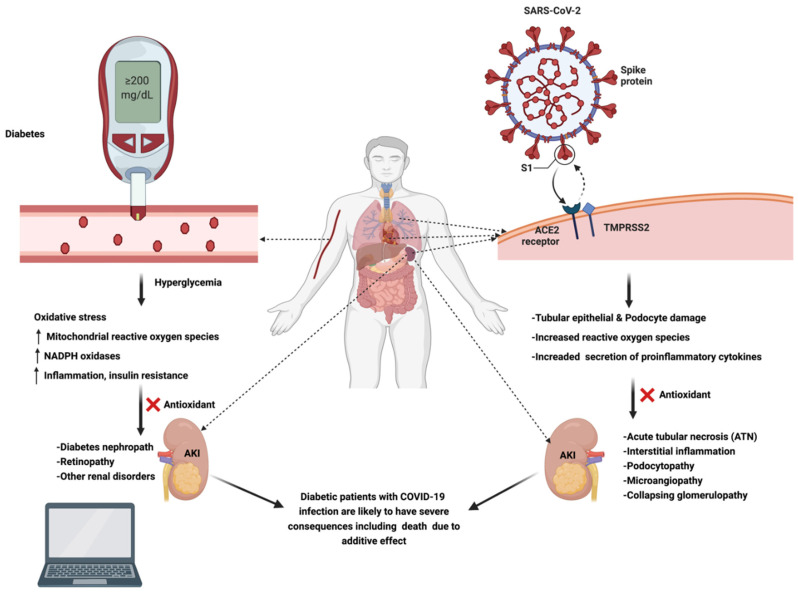
SARS-CoV-2 and diabetes induce kidney damage via oxidative stress: the role of antioxidants. Abbreviations: ↑: increase; CKD, chronic kidney disease AKI, acute kidney injury; SARS-CoV-2, severe acute respiratory syndrome coronavirus-2; COVID-19, coronavirus disease 2019; TMPRSS2, Transmembrane protease serine 2. (The figure was made with www.biorender.com, accessed on 13 December 2021).

Both diabetes and COVID-19 cause oxidative damage and inflammation in tissues and share common molecular pathways to generate clinical symptoms. As discussed, the presence of both diseases, COVID-19 and diabetes can cause synergistic oxidative stress, severe inflammation, vasoconstriction, and thrombosis in capillary blood vessels, mainly in the kidney and lungs, and therefore cause synergistic damage in these organs that leads to death. A study conducted on 174 COVID-19 patients (24 patients among them diabetic) found that diabetic patients with COVID-19 were at an increased risk of poor prognosis due to higher risks of severe pneumonia and out-of-control inflammatory responses [43]. Another study reported that the chance of developing COVID-19 pneumonia is 87.9% higher in patients with diabetic nephropathy, and the probability of ventilation is 101.7% higher, probability of a fatal outcome is 20.8% more compared to chronic kidney disease alone [44]. Noticeably in this regard, a recent study found significantly lower mortality in metformin-administered COVID-19 diabetic patients (3/104, 2.9%) than in the non-metformin-administered COVID-19 diabetic group (22/179, 12.3%, *p* = 0.01), suggesting that blood sugar control is a significant factor in reducing mortality rates when diabetes is a comorbid factor with COVID-19 [45]. However, metformin can act through a secondary mechanism. Since the drug acts through AMPK (AMP-activated protein kinase) activation, such activation can lead to phosphorylation of ACE2, the receptor for SARS-CoV-2 [46]. This in turn can lead to conformational and functional changes in ACE2 leading to decreased binding ability of the SARS-CoV-2 spike protein receptor binding domain (S-RBD), leading to decreased entry of the virus into human host cells. The presence of a large phosphate moiety on ACE2 due to phosphorylation by AMPK can further be a factor in decreased binding ability of S-RBD to ACE2 because of steric hindrance [46]. Furthermore, COVID-19 can by itself cause AKI, a fact recognized earlier on following the outbreak of the pandemic [47]. We hypothesize that COVID-19 and diabetes increase oxidative stress that can play a synergistic role in damage to the kidneys, when present as comorbidities (Figure 2) [48,49].

Interestingly, some antioxidants like flavonoids have been suggested as a complementary therapy for COVID-19 [50] and diabetes [51], which could be beneficial in ameliorating kidney damage during COVID-19 infection with diabetes as a comorbidity [52]. For example, the flavonoid apigenin reportedly attenuated renal dysfunction, oxidative stress and fibrosis in streptozotocin-induced diabetic rats [53]. Apigenin has also been shown in in silico studies to be an inhibitor of Mpro, the main protease of SARS-CoV-2 and which plays a vital role in viral replication [54]. Apigenin is not the only example of its type. The flavonoid quercetin reportedly acts as a prophylactic to COVID-19 [55,56], as well as an antidiabetic and antioxidant compound. Moreover, recently, a preclinical study showed quercetin’s renal protective effects [57]. Intragastric administration of quercetin (1.5 and 3 g per kg body weight daily for eight weeks) effectively reduced apoptosis of renal cells and plasma levels of blood urea nitrogen, creatinine, and uric acid in male Sprague Dawley rat model of chronic renal failure [57]. The study also reported that quercetin treated rats showed reduced inflammation by preventing phosphoinositide 3-kinase (PI3 k)/Akt (protein kinase B) signaling pathway by targeting phosphoinositide 3-kinase regulatory subunit 1 (PIK3 R1) and reduced expression NLRP3, p-PI3 k, Phospho-Akt (p-Akt), and caspase1 in kidney tissues [57]. Another study reported that in a mouse model of renal dysplasia, quercetin treatment increased the epithelial organization of developing nephrons, inhibited nuclear beta-catenin, and thus improved renal dysplasia [58]. A report showed that combined pretreatment of 30 mg/kg resveratrol and 50 mg/kg quercetin over a period of seven days prevented paracetamol-induced (2 g/kg body weight) acute renal failure via reducing plasma creatinine, urea, and inflammatory markers (e.g., MDA, IL-6, and TNF-α) [59].

Modlinger and colleagues show that oxidative stress can cause salt retention in kidneys by promoting the expression of vasoconstrictor molecules and NADPH oxidase, and thus it can cause acute to chronic renal failure [60]. Another report mentioned that COVID-19 causes activation of the innate immune response and secretion of inflammatory cytokines due to the development of oxidative stress [61]. The cytokine storm seen repeatedly in COVID-19 patients has been hypothesized to be a consequence of oxidative stress [50]; as such, it can be expected that antioxidants such as flavonoid compounds would relieve COVID-19 severity, similar to antioxidant flavonoid effects on ameliorating diabetic cardiac myopathy through alleviation of oxidative stress [62] and diabetic nephropathy through a similar mechanism. Quercetin, apigenin, baicalin, luteolin, hesperidin, genistein, proanthocyanidin and eriodictyol have been found to be capable of alleviating oxidative stress in diabetic nephropathy [63]. Incidentally, all the above flavonoid compounds have been reported to bind to SARS-CoV-2 protein components or the receptor hACE2 [54,55,56,64,65,66,67,68,69]. These flavonoids are also antioxidants suggesting a common mode of action in both COVID-19 and diabetes, which in all probability is through reducing oxidative stress.

There are also recommendations on using Chinese herbal medicines and polyphenolic compounds containing antioxidants as an adjuvant to reduce the severity and mortality of COVID-19 patients with diabetes [70,71]. Besides flavonoids, phenolic compounds, which have antioxidant capacity and are present in essential oils of plants, may play a similar beneficial role in reducing oxidative stress during diabetes and COVID-19. Eugenol, a phenolic compound present in clove (*Syzygium aromaticum* (L.) Merr. & L. M. Perry, family: Myrtaceae), has been shown to ameliorate insulin resistance, oxidative stress, and inflammation in high fat diet/streptozotocin-induced diabetic rat [72], inhibit pancreatic α-amylase [73], and inhibited α-glucosidase activity and formation of advanced glycation end-products [74]. Antioxidant therapy prevented the cardiovascular disorders of patients who require dialysis, but the effect was not seen in patients with chronic kidney disease (CKD). Importantly, Jun and colleagues reported that antioxidants could reduce the development of kidney disease (late-stage) and serum creatinine levels by improving serum clearance of creatinine. The study reported that antioxidant therapy did not increase life-threatening adverse events, indicating its possible safety, although it needs validation from a larger population cohort and more comprehensive observational studies [75].

A recent RCT investigated the effect of 1 g of quercetin (along with standard care) over a period of four weeks in COVID-19 patients (*n* = 76, per group) and observed reduced severity of COVID-19 symptoms, duration of hospitalization, artificial ventilation, and fewer deaths in comparison with patients with standard care (without quercetin supplementation) [76]. Another pilot RCT from the same group of authors found that 600 mg of quercetin supplement over a period of 2 weeks improved COVID-19 related clinical symptoms and relevant plasma parameters on a small number of patients and compared against standard care group (*n* = 21) [77]. On the other hand, another RCT did not observe any effect of the antioxidant, ascorbic acid on a small number of COVID-19 patients (*n* = ~53 each group) treated over a period of 10 days with ascorbic acid (8 g), zinc gluconate (50 mg), or both agents, and none (standard of care) [78]. Similarly, a second RCT with 6 g/day (1.5 g, four times daily) intravenous ascorbic acid supplement with standard care for 5 days produced no improvement against patients with standard care (*n* = 30 per group) [79]. Another RCT planned to administer 24 g/day vitamin C for 7 days intravenously on COVID-19 patients but finished the study without reporting any results [80]. From these limited numbers of available clinical trials, the reports were based on small numbers of patients. More extensive studies are required over an extended period to make any fruitful comment on the effectiveness of these antioxidant compounds against SARS-CoV-2.

Molecular docking studies showed that the compound (quercetin) has high binding affinities to various targets in SARS-CoV-2 [81], and can be a potential nutraceutical against COVID-19 [82,83,84]. It is evident that both diabetes and COVID-19 induce the over-production of reactive oxygen species, which ultimately may cause damage to many vital organs, including the kidney, heart, and lungs [17]. It is also evident from some studies that antioxidants can reduce kidney disease. There are increased hospitalization and mortality rate with COVID-19 patients with diabetes. It is hypothesized that antioxidant therapy may reduce the fatality of COVID-19 patients with diabetes by reducing the over-production of the reactive oxygen species. However, this concept is in an early stage and needs many studies to validate this concept. The case can then be made for antioxidants (flavonoids and phenolic compounds) for use as therapeutic or nutraceutical in the case of COVID-19 patients and who have diabetes as a comorbidity for these compounds antioxidative capacities (Table 1).

Various in silico studies demonstrated that quercetin, luteolin, myricetin, naringenin, and hesperidin could interfere with various stages enzymes of SARS-CoV-2 (viral papain such as protease (PLpro) [85], and main protease (Mpro; 3 CLpro, also named 3-chymotrypsin-like protease) [92,97], NTPase/helicase) [99] of the coronavirus entry and replication cycle [85,92,95,97]. On the other hand, kaempferol [89], luteolin [92], apigenin [97], and catechin-like flavonoids [99] interact and inhibit (in silico) SARS-CoV-2 spike proteins (especially S2) and hACE-2 receptors, and thus can prevent viral entry inside the host cells [89,92,97,99] (Table 1).

It is noticeable that most of the antioxidant activities of flavonoids were measured (Table 1) using chemical reactions and assessing the kinetics or reaching the equilibrium state such as free radical scavenging activity against 2,2′-Azinobis-(3-ethylbenzothiazoline−6-sulfonic) acid (ABTS) and [2,2-di(4-tert-octylphenyl)-1-picrylhydrazyl] (DPPH) free radicals, as these reagents cause oxidative stress (overproduction of reactive oxygenated species, ROS) [101]. The main issue is that normal cells produce small amounts of ROS, which cannot be measured correctly using current colorimetric methods. Noticeably, some of these flavonoids were tested for antioxidant enzyme activities such as superoxide dismutase, glutathione peroxidase, and catalase enzymes in pancreatic cells using a STZ-induced diabetic rat model [87,102]. It needs to be further pointed out that flavonoids do not just reduce oxidative stress through scavenging of free radical species but also through inhibition of ROS producing enzymes such as xanthine oxidase [85] or through chelation of metal ions [87].

Flavonoids showed antidiabetic effects, such as quercetin inhibited glucose absorption from intestine, improved glucose use from peripheral tissues, as well as it simulated insulin secretion. Studies also suggest that consumption of quercetin displayed a long plasma half-life in humans [103]. Furthermore, a meta-analysis on the effects of quercetin showed that the flavonoid reduced blood glucose levels in a dose-dependent manner in experimentally induced (e.g., STZ-induced) diabetic animals, and it is effective at higher doses (10, 25 or 50 mg/kg body weight) [104]. Quercetin inhibits the enzymes dipeptidyl peptidase IV (DPP-IV) and thus shows antioxidant and antihyperglycemic properties [105]. Importantly, it is a generally recognized as a safe compound according to FDA [106]. Quercetin also inhibited TNF-α-mediated inflammation and insulin resistance in human adipose cells in an in vitro study [107]. Another flavonoid, kaempferol increases glucose uptake and glucose transporter 4 translocation via a Janus kinase 2-dependent pathway in skeletal (L6) myoblast cell line, which indicates kaempferol’s hyperglycemic effect in vitro [108]. A clinical study showed that consumption of a formulation that contained myricetin, quercetin, chlorogenic acid (another group of polyphenol compounds) reduced plasma glucose levels in confirmed diabetes-2 patients, and cotreatment with metformin showed potentiation of metformin’s antidiabetic activities [109]. Three times daily application of a topical formulation contained quercetin for four weeks improved numbness, jolting pain, and irritation, and quality of life of patients who experience symptomatic diabetic peripheral neuropathy in a small number of patients (total *n* = 34) [110]. Another clinical trial showed no effect of a flavonoid against placebo over a 12-week combined treatment of isoquercetin (225 mg once daily) and sodium nitrite (40 mg twice daily) in CKD patients (*n* = 35 per group) [111]. Noticeably, an antioxidant such as resveratrol (a stilbenoid compound) caused suppression of angiotensin-2 that may be used as an adjunct therapy to COVID-19 [112,113]. It seems that not all antioxidants are effective in preventing oxidative stress. The capability of preventing oxidative damage varies between compounds, which needs further extensive clinical trials to elucidate the efficacies of these compounds.

Vitamin D (a natural antioxidant) and magnesium deficiencies also exacerbate the underlying pathogenetic mechanisms in COVID-19 [114]. Vitamin D is essential to maintaining a healthy immune system [115]. Vitamin D levels were shown to be associated with blood glucose and body mass index of COVID-19 patients. As suggested by di Filippo and colleagues, a common pathophysiological mechanism might be involved with hyperglycemia, adiposity, and COVID-19 severity [116]. Magnesium activates vitamin D and protects cells from oxidative stress [114]. Severe COVID-19 patients showed lower vitamin D levels and higher oxidative stress parameters (like plasma LDH, peroxides, and oxidative stress index) than less severe COVID-19 patients [117]. A randomized clinical trial in Spain, oral supplement of calcifediol (25-hydroxyvitamin D3: 0.532 mg on day 1, 0.266 mg on days 3 and 7, and weekly afterwards) in COVID-19 patients (*n* = 50) along with standard treatment for COVID-19 in hospital reduced the severity of symptoms and admission to Intensive Care Units (ICU) than standard care group (*n* = 26) [118]. As the study was based on a small number of COVID-19 patients (total *n* = 76) and there was in-equality of sample sizes between control and treatment groups, the study requires further validations to comment on the efficacy of vitamin D against COVID-19. However, it is re-ally a promising study that antioxidants such as calcifediol helped reduction of COVID-19 severity and ICU admission [118]. Noticeably, people with inherited glucose-6-phosphate dehydrogenase (G6 PD) deficiency can cause of reduced circulatory 25-hydroxyvitamin D in blood, and can be vulnerable to excess oxidative stress, cytokine release, and pulmonary dysfunction due to COVID-19 infection [119]. It is important to note that there is no strong clinical evidence for flavonoids or vitamin C against protection from oxidative damage caused by COVID-19. Vitamin D can prevent oxidative damage produced by SARS-CoV-2 in people suffering from COVID-19. However, further evidence is required in larger population cohorts based on various geographical locations, age groups, food habits, and ethnicity.

**Figure 3:**
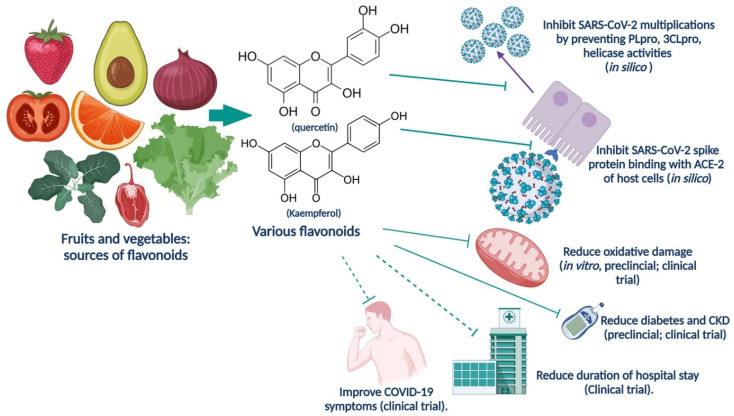
Roles of flavonoids against SARS-CoV-2 based on recent in silico, pre-clinical, and clinical studies. Abbreviations: CKD, chronic kidney disease; SARS-CoV-2, severe acute respiratory syndrome coronavirus-2; COVID-19, coronavirus disease 2019. (The figure was made with www.biorender.com, accessed on 13 December 2021).

Various fruits and vegetables are sources of flavonoids. Common vegetables such as tomatoes are natural sources of quercetin, kaempferol, and naringenin [120] (Figure 3). Broccoli, celery, cabbages, peppers, and parsley are sources for luteolin [121,122]. Noticeably onions and tea are main dietary sources of flavonols (e.g., quercetin and kaempferol) and flavones (apigenin and luteolin) [123]. Onions, parsley, sage, tea, citrus fruit (like oranges, lemons, and limes), apples, grapes, cherries, and berries are potential sources of quercetin and other flavonoids [123,124,125,126] (Figure 3). Noticeably parsley, onion, zinger (source of hesperidin), citrus fruit-peels, sage are sources of essential oils, which can improve the bioavailability of flavonoids (like quercetin microemulsion of peppermint, clove and rosemary oils) [127]. Essential oils are sources of antioxidants, improve the quality of life of diabetic patients, analgesics, and may have the capability to improve COVID-19 and related comorbidities [128,129,130,131,132]. Iddir and associates reported that poor nutrition stimulates increased oxidative stress and inflammation, which render poor immunity against pathogens. However, dietary protein intake can help antibody production, and micronutrients such as vitamins D, A, C, and E, flavonoids, carotenoids, and minerals such as zinc can prevent the expression of transcription factors (NF-kB and Nrf-2) related to inflammation [133]. This information is also supported by a clinical study with COVID-19 patients that showed reduced plasma antioxidant levels than people without SARS-CoV-2 infection [39].

## 6. Conclusions

COVID-19 and diabetic patients have a common feature of increased oxidative stress. Patients with both disorders generally end up with poor prognosis and death. A large part of this poor prognosis and death is caused by kidney failure. COVID-19 and diabetes may both be responsible by increasing oxidative stress in a synergistic manner. Flavonoids and polyphenols, because of the nature of their chemical structure are good antioxidants. These phytochemicals can scavenge reactive oxygen species (ROS) and inhibit enzymes responsible for making ROS. They also inhibit production of ROS through chelation of metal ions. We suggest that this oxidative stress factor of COVID-19 with diabetes as a comorbidity and vice versa has been overlooked largely. We further recommend that judicious use of vitamin D, flavonoids, and other antioxidants as possible therapeutics, may mitigate this oxidative stress effect and improve the prognosis of patients suffering from both COVID-19 and diabetes.

## Figures and Tables

**Table 1:** Several dietary flavonoids with anti-COVID-19, antioxidant and antidiabetic properties.

**Flavonoid**	**Anti** **-** **COVID** **-** **19**	**Antioxidant**	**Antidiabetic**
Quercetin	In silico and in vitro studies demonstrated that quercetin can interfere with various stages of the coronavirus entry and replication cycle such as PLpro, 3CLpro, and NTPase/helicase [85,86].	Significantly increased antioxidant enzyme activities in streptozotocin (STZ)-induced diabetic rats [87]. DPPH and ABTS radical scavenging activities reported [88].	Pre-treatment prevented STZ-induced diabetes in rats [87].
Kaempferol	In silico studies showed that kaempferol can inhibit Spike glycoprotein of SARS-CoV-2 [89].	DPPH and ABTS radical scavenging activities reported [88]. Antioxidant effect observed in DPPH (2,2-diphenyl-1-picrylhydrazyl), ABTS^+^ radical scavenging and xanthine oxidase inhibition assays [90].	Dipeptidyl peptidase IV (DPP-4) and α-glucosidase inhibitory effect was observed [90].
Myricetin	Inhibition of SARS-CoV-2 replication by targeting Mpro (in silico) and ameliorating pulmonary inflammation (reducing bleomycin-induced pulmonary inflammation in mice) [91].	Antioxidant effect observed in DPPH (2,2-diphenyl-1-picrylhydrazyl), ABTS^+^ radical scavenging and xanthine oxidase inhibition assays [89].	Dipeptidyl peptidase IV (DPP-4) and α-glucosidase inhibitory effect was reported [89].
Luteolin	In silico studies show luteolin to bind strongly to Mpro, PLpro, and ACE-2 [65]. In silico studies indicated that luteolin can bind to S2 unit of spike protein (S) of SARS-CoV-2 [92].	DPPH and ABTS radical scavenging activities reported [88].	Luteolin ameliorated diabetes in mice. Luteolin improved blood glucose, HbA1c (hemoglobin A1c), and insulin levels. Anti-inflammatory and anti-oxidative effects of luteolin were also observed [93].
Apigenin	In silico studies indicated that apigenin can bind to S2 unit of spike protein (S) of SARS-CoV-2 [92].	DPPH and ABTS radical scavenging activities reported [88].	The beneficial roles played by apigenin in diabetes mellitus have been reviewed. The compound is an antioxidant; metabolism of glucose and transfer to peripheral tissues are enhanced; pancreatic secretion of insulin is increased; activities of gluconeogenic enzymes and aldose reductase enzyme are suppressed leading to prevention of diabetic complications like cataract, retinopathy, and neuropathy [94].
Naringenin	In silico evidence of Mpro inhibition and reduction of angiotensin-converting enzyme receptors activity, reviewed by Tutunchi et al. [95].	Antioxidant and anti-diabetic effects observed in STZ-nicotinamide-induced diabetic rats as shown by significantly lower mean levels of fasting blood glucose and glycosylated hemoglobin, significantly elevated serum insulin levels, significantly higher mean activities of pancreatic enzymatic antioxidants, significantly higher mean levels of plasma non-enzymatic antioxidants, lower mean pancreatic tissue levels of MDA and lower mean activities of alanine aminotransferase (ALT), aspartate aminotransferase (AST), alkaline phosphatase (ALP) and lactate dehydrogenase (LDH) in serum [96].	Antioxidant and anti-diabetic effects observed in STZ-nicotinamide-induced diabetic rats as shown by significantly lower mean levels of fasting blood glucose and glycosylated hemoglobin, significantly elevated serum insulin levels, significantly higher mean activities of pancreatic enzymatic antioxidants, significantly higher mean levels of plasma non-enzymatic antioxidants, lower mean pancreatic tissue levels of MDA and lower mean activities of alanine aminotransferase (ALT), aspartate aminotransferase (AST), alkaline phosphatase (ALP) and lactate dehydrogenase (LDH) in serum [96].
Hesperidin	In silico studies indicate that hesperidin may bind to multiple components of SARS-CoV-2 (like Mpro, PLpro, Spike protein) and its human receptor ACE2, reviewed by Agrawal et al. [97].	Antioxidant and anti-diabetic effects observed in nicotinamide-STZ-induced diabetic rata [98].	Antioxidant and anti-diabetic effects observed in nicotinamide-STZ-induced diabetic rata [98].
Catechin	As shown in in silico studies, catechin can bind to S protein of SARS-CoV-2 and hACE2, thus inhibiting viral entry [99].	Catechin showed antioxidant activity such as free radical scavenging activity against DPPH and ABTS free radicals [100].	Catechin inhibited activity of α-amylase and α-glucosidase; catechin also significantly decreased the different lipid parameters, hepatic, and renal function enzyme levels along with Hb1c level in diabetic rats [100].

Abbreviations: ACE-2, angiotensin-converting enzyme 2; DPPH, 2,2-di(4-tert-octylphenyl)-1-picrylhydrazyl; PLpro, papain-like protease; 3CLpro, 3-chymotrypsin-like protease; NTPase, nucleoside-triphosphatase; Mpro, main protease; HbA1c, hemoglobin A1c; DPP-4, Dipeptidyl peptidase IV; STZ, streptozotocin; ABTS, 2,2′-azinobis-(3-ethylbenzthiazolin-6-sulfonic acid); SARS-CoV-2, severe acute respiratory syndrome coronavirus-2; COVID-19, coronavirus disease 2019.

## Data Availability

Not applicable.
